# Chairman’s Address

**Published:** 1903-09

**Authors:** Dunbar Roy

**Affiliations:** Atlanta, Ga.


### CHAIRMAN'S ADDRESS.*

By DUNBAR ROY, M.D.,

ATLANTA, GA.

This has well been called the "strenuous age." There is no profession, cult or commercial business which has not felt the touch of this strenuosity. The times call for progressiveness in every line of work, and he who is indolent will soon be gliding down the stream of life with but few congenial friends. While this spirit is dominant in every avocation of life, it is especially recognized in its relation to the practice of the healing art.

Competition in business is well seen in every line of commercial and professional life. We hear much of the "overcrowded pro-

*Read before the Southern Section of the American Laryngological, Rhinological and Otological Society.

fession," which in truth is no fact of idle fancy. Year by year the ranks of the medical profession are augmented by additions from the too numerous medical colleges. All must live, and too soon this one fact often causes the physician to allow deceptive methods to creep into the regular routine of his work. I have often thought that if there was one specialty which required honest, upright and truthful men, it was in the one represented by those of us who have assembled here to-day.

While our program is short, we must congratulate ourselves upon its quality. It is indeed a pleasure to number with us to-day several of our confrères who have come so long a distance to aid in making this meeting of the Southern Section a success. Unfortunately we live in a country of beautiful distances, and personally I feel it a very high compliment to have these gentlemen with us to-day, representing as they do different sections of our country. Our southern representation in this national society is as yet small, but like the country in which we live, the future only can tell of its possibilities.

I believe I may truthfully say that specialism too often causes us to be narrowed in the horizon of our clinical work, and too infrequently do we stop to consider those broad principles of medicine which should be utilized in the management of every case. In other words, we become too prone to treat nose and throat diseases as a local trouble to be remedied by local applications, when quite often the cause is some systemic dyscrasia far removed from the seat of supposed trouble. There are many workers to-day in the field of rhinology who would have us treat the nasal cavities as we would a mathematical proposition—to be governed by fixed and invariable rules. They would teach the student of medicine that there must be a fixed diameter of the nasal cavities, to be made so if necessary by wholesale destruction of tissue. They seem to forget that the divine Creator has never made a perfect man, and we might say perfectly proportioned nasal cavities. They believe, that because one side of the nose is smaller than the other, that such an individual must be subjected to months of local treatment in attempts to make the two sides symmetrical. With increasing clinical experience, I believe that the conscientious and painstaking rhinologist will find that systemic disturbances play even a more important rôle in nasal affections than the so-called local anomalies. Hence a plea I have always held is for a previous general hospital experience before the study of a specialty, or even several years confined to general practice. In daily routine of work, looking at only one portion of the anatomy, we are so apt to attach undue importance to some local anomaly and forget to study other conditions existing in the human frame.

Take, for instance, stenosis of the nasal passages, a condition so prevalent among a large majority of people who live in crowded cities where dust and fumes permeate the atmosphere, and where good ventilation is often at a premium. In such cases we see the cause to be due to external surroundings, producing nasal symptoms even when these cavities show no distinct pathological changes.

Take again the plethoric heavy drinker, or even persons of convivial habits who sit up late and are unsystematic in their habits. Such people are troubled almost universally with nasal stenosis, especially in the early morning hours. Here we have causes which may be classed as internal irritants, which by their removal will, in the large majority of cases, cause a cessation of the nasal symptoms. In fact my observation leads me to the conclusion that purely local pathological conditions constitute but little over one half of the causes which compel the patient to seek the services of the rhinologist.

This aggressive treatment of nasal diseases is much to be deplored. I mean by aggressive such things as the too frequent use of the electro-cautery, the operative intervention for every anomaly which prevents the nasal chambers from being mathematically correct in their proportions, the daily use of sprays which is unscientific and the producer of an exceedingly pernicuous habit—these and kindred modes of treatment, when carried to extremes, are not far from being more detrimental than no treatment whatever.

The electro-cautery has a valuable place as a therapeutic remedy in nasal diseases, but I am confident that it has been much abused.

In true hypertrophic condition of the turbinates, it is often a remedy which can not be equalled, but for it to be used in those cases of passive engorgement of the nasal mucous membrane or where all enlargement disappears under the application of cocain, is bad therapeutic judgment. Many physicians starting out as rhinologists do much injury to the nose by the use of the electro-cautery before their clinical experience and observation has taught them their mistakes. Many of us have no doubt seen nasal synechias as the result of the injudicious use of the cautery. Curtis, in a paper before the New York Academy of Medicine, as did also several other speakers on that occasion, deprecates the use of the cautery on the nasal septum, an observation which coincides with my own experience, except of course in rare instances. Furthermore the use of the cautery has not proven to be entirely free from danger, for Dr. Levy, of Denver, in a paper before this Association, recites a number of cases of meningitis, sinus thrombosis and the like, which resulted from the use of the cautery upon the turbinates.

The sooner we recognize the fact that all swollen conditions of the turbinates do not need local treatment, especially the cautery, the better it will be for our patients.

A very significant paper, by Dr. Mulford, of Buffalo, was published in the *Laryngoscope* a short time ago entitled "The Importance of Urinalysis to the Laryngologist." In this paper a number of cases were recorded where the turbinates were seemingly hypertrophied and where the condition entirely disappeared under treatment directed to the kidneys. I believe that we, as rhinologists, should institute a more thorough general examination before the application of any local therapeutic. Dr. Mulford makes this significant remark in the paper referred to, which agrees entirely with my own clinical experience. He says: "In individuals subject to frequent colds, there will be found always some fault in the body functions, and that fault will usually be shown in the urine."

What I have said in regard to the use of the electro-cautery applies with equal force to the indiscriminate removal of septal spurs which are almost universally present. It is the height of folly to say that every septal spur should be removed, just as it would be to say that the Gasserian ganglion should be extirpated in every case of facial neuralgia.

A certain Western rhinologist who is rather verbose in his writings, and rather fond of euphonious titles, would have the profession taught that every nasal chamber should be placed in mathematically correct proportions. This same author would have absolutely no touching between the septum and any of the turbinates, with the result that there would be no individual living who does not need treatment. This idea carried out in a practical way would certainly fatten our pocketbook, especially if we could make the patient submissive to these views.

This same writer says: "As a routine practice . . . . it is my custom to prepare the parts by prescribing hourly use of a mild alkaline cleansing solution for a period of a week or ten days." Think of an individual excusing himself every hour to take a douche. If this is not carrying the treatment of nasal diseases to extreme absurdity, then my bump for comprehending the ridiculous is poorly developed.

I believe we should call a halt upon such ridiculous teaching as the above if we do not want the public to view us in the light of thieving specialists.

While it is true that the mucous membrane of the nose is by no means susceptible to septic infection as a result of intra-nasal surgery, it is, however, necessary for us to use proper precaution to prevent such accidents in every case which comes under our professional care. I believe that the extremists are doing much injury to the profession to-day, and such should not receive our unanimous commendation. It is sometimes easy perhaps to satisfy our conscience into the belief that a septal spur is the cause of some supposed nasal disturbance when we remember that a patient is very much more willing to pay a good fee for an operation than to pay one for a prescription.

Fortunately a saner reaction is taking place among rhinologists, and in the future we believe there will be much less destruction of the nasal mucous membrane by unsound surgical procedures than has heretofore been the case.

To quote from a recent address of Dr. Chas. H. Knight before the American Therapeutic Society: "In consequence of this rhinological enthusiasm, the nose has been looked upon as a probable source of nearly every human ill, and a ruthless slaughter of intra-nasal structures, without regard to their physiological functions, has been universally practiced. Happily, a saner view is coming to prevail, and the belief is growing that epilepsy, asthma, dysmenorrhea and arthritis of the knee may have other causes than a hysterogenic zone or a point of pressure within the nasal fossæ."

In using the word conservatism, I would not limit its application to surgical procedures within the nasal cavities, but would ask for a wider range of its application.

Two years ago I read a paper upon "The Use and Abuse of Nasal Sprays," and a plea for their more rational application. The more extended becomes my observation, the more am I convinced that nasal sprays are too frequently injudiciously used.

Three ways are they more especially abused;

(1) By the character of the medicinal substance used. (2) By the force of the spray. (3) By the frequency of their use.

The spray habit is no idle fancy, for inquiry will elicit the fact that there are numerous people who not only use a spray containing cocain, but one also containing an oily menstruum with menthol, both of which, in the long run, will prove injurious to the nasal structures.

The habit of going to a specialist to be "sprayed out" is indeed a money-making one for the former, but exceedingly injurious to the latter.

The time has come when we should call a halt upon this pernicious habit if we would not see an honorable specialty debased to the level of commercial charlatanism.

These few rambling remarks have, however, gone beyond what should be the limit of the Chairman's address, and in closing I have the pleasure to call upon others who have so kindly aided in the formation of this excellent program.

*Grand Opera House.*

